# Impact of Obesogenic Environments on Sugar-Sweetened Beverage Consumption among Preschoolers: Findings from a Cross-Sectional Survey in Beijing

**DOI:** 10.3390/nu14142860

**Published:** 2022-07-12

**Authors:** Ruijie Yan, Enying Gong, Xinxuan Li, Lutong Zheng, Wei Liao, Kaiyuan Min, Fenghua Su, Lianjun Wang, Jing Wang, Denghui Hu, Yuxiang Tang, Juan Zhang

**Affiliations:** 1 School of Population Medicine and Public Health, Chinese Academy of Medical Sciences and Peking Union Medical College, Beijing 100730, China; yanrj@sph.pumc.edu.cn (R.Y.); gongenying@cams.cn (E.G.); hu_dhui@163.com (D.H.); tyxxkai@163.com (Y.T.); 2 School of Health Humanities, Peking University, Beijing 100191, China; lixinxuan@pku.edu.cn (X.L.); zhenglt@pku.edu.cn (L.Z.); 3 Institute of Basic Medical Sciences, Chinese Academy of Medical Sciences and Peking Union Medical College, Beijing 100730, China; liaowei@pumc.edu.cn (W.L.); min_kaiyuan@163.com (K.M.); 4 Dongcheng Center for Disease Control and Prevention, Beijing 100013, China; sufh0529@163.com (F.S.); wlj63@163.com (L.W.); jing2000@sohu.com (J.W.); 5 Center of Social Medicine, National Research Institute for Family Planning, Beijing 100081, China

**Keywords:** sugar-sweetened beverages, preschoolers, obesogenic environments, caregivers

## Abstract

The excessive consumption of sugar-sweetened beverages (SSBs) has been proven to be critical for obesity among preschoolers. This study aimed to describe the SSB consumption rates among preschoolers in the Dongcheng District of Beijing, China, and to explore the association between obesogenic environmental determinants and consumption. We applied a stratified cluster sampling method and recruited 3057 primary caregivers of preschoolers in June 2019 to participate in the survey. The caregivers reported their children’s consumption rates of six categories of SSBs and their exposure rates to SSB-related obesogenic environments. The associations between them were tested using multivariate logistic regression models. The mean (SD) age of the children was 5.6 (0.6) years and nearly half (48.3%) were girls. About 84.5% of the children had consumed SSBs over the past three months, and sugar-sweetened milk beverages had the highest consumption rate. Higher exposure to advertisements for the corresponding SSB categories in children, higher frequency rates of consuming SSBs and of taking children to fast-food restaurants in caregivers, and lower frequency rates of reading the Nutrition Facts Panels by caregivers were associated with higher SSB consumption rates among children (*p* < 0.05 in all of the SSB categories investigated, except for the Nutrition Facts Panel reading behaviors for the sports and energy beverages). SSB consumption among preschoolers is of concern, and comprehensive policy actions and education are urgently needed.

## 1. Introduction

Childhood obesity is a growing global public health challenge [1], which can deteriorate into obesity when these children grow up and which places them at increased risk along the life continuum [2,3], i.e., for poor self-esteem, depression, discrimination, type 2 diabetes, hypertension, hyperlipidemia, and various cardiovascular diseases. In China, the prevalence of overweight and obesity in children under six years old in 2015–2017 was 10.4% (approximately 10 million) [4,5].

The root cause of overweight and obesity is an imbalance between the energy input and the energy output. Foods that are high in calories and low in nutritional value are the main sources of excessive energy, among which sugar-sweetened beverages (SSBs) have emerged as the primary contributors of added sugar intake among Chinese children [6]. Prospective cohort studies and clinical trials have provided strong evidence of the relationship between SSBs and weight gain and the risk of related chronic diseases [7]. Numerous evidence-based recommendations have been proposed to limit SSB consumption internationally [8,9], and the Chinese Dietary Guidelines (2022) from the Chinese Nutrition Society also advises preschoolers in China to drink more water and avoid SSBs [10]. However, a previous study revealed a rising trend in the SSB sales volume per capita in China from 2000 to 2014 [11], with 75% of children aged 4–9 years consuming  ≥  1 serving of SSBs/week in 2016 [12]. In addition, based on the data collected in a previous study between 2013 and 2018, the beverages in China had the highest median total sugar content as compared with many other countries, such as Australia, Canada, Chile, the UK, and the USA [13]. The existing evidence suggests that the intake of SSBs in early childhood was significantly associated with higher SSB intake later in life [14]. Therefore, it is urgent to take effective measures to alter this situation.

Young children are often exposed to circumstances in which they may not voluntarily participate or have minimal control [15,16]. Their SSB consumption could be affected by their families, kindergartens, beverage manufacturers, and government-related determinants [17]. Parental lower socioeconomic status, lower age, SSB consumption, using food as rewards, living near a fast-food or convenience store, and school nutrition policies and staff skills were proven to be well-grounded determinants across other countries [17,18]. Meanwhile, the consistent evidence suggests further exploring the potentially modifiable obesogenic environmental determinants of SSB marketing, advertising across all channels, and nutritional labeling [19].

Given the extensive evidence of SSBs contributing to severe health consequences [20,21,22,23], few studies have investigated the correlates of SSB intake among children in China with different food environments. The limited current evidence in China is mainly focused on school-aged children and the factors at the individual level [24,25,26]. To fill in the gaps in the scarce evidence for preschoolers, this study investigated the SSB consumption among preschoolers in the Dongcheng District of Beijing, China, and explored multifaceted obesogenic environment determinants of children’s SSB consumption.

## 2. Materials and Methods

### 2.1. Study Population

This study used data collected in June 2019 from a repeated cross-sectional study [27] that aimed to assess obesity and related behaviors among preschoolers in local kindergartens in the Dongcheng District of Beijing. The study was approved by the Ethics Committee of the Dongcheng Center for Disease Control and Prevention (DCCDPCIRB20180416–1). All caregivers gave their written informed consent.

At the stage of recruitment in the initial survey in 2018, we applied a stratified proportionate cluster sampling for a representative sample in the Dongcheng District of Beijing. As many preschoolers could not read and write, one of their primary caregivers (hereafter referred to as “caregivers”) was eligible to participate in the survey on their behalf. We recruited caregivers with the help of teachers in charge of each kindergarten class. The inclusion criteria were caregivers of children in the first and second grades of the chosen fifteen kindergartens with written informed consent. A detailed description of the sampling procedures was published elsewhere [27].

In the present survey, one kindergarten refused to be involved, and all caregivers of the 3595 children in the second and third grades from the other fourteen kindergartens were eligible and invited to participate, accordingly. The participants in the present survey slightly differ from the initial survey in these fourteen kindergartens because some students transferred in and out during this period.

### 2.2. Measurements

To obtain SSB consumption frequencies and other essential variables, the teachers in charge of each class handed out questionnaires with unique ID numbers to caregivers. All teachers in this study received systematic training and provided necessary instructions to the caregivers. The caregivers then completed the online questionnaires anonymously.

#### 2.2.1. Outcome: Children’s SSB Consumption

We adapted a validated food frequency questionnaire [28] to measure the children’s consumption in the past three months for six SSB categories most frequently consumed by Chinese children, with seven Likert levels ranging from “never” to “more than seven times per week”. We also annotated several contextual examples for SSB categories to improve the interpretability [29]: sugar-sweetened milk beverages that were not milk or yogurt (hereafter referred to as “milk beverages”, e.g., lactobacillus beverages, Nutri-Express, AD Calcium Milk), vegetable- or fruit-flavored beverages that were not 100% fruit or vegetable juice (hereafter referred to as “fruit/vegetable beverages”, e.g., Orange Multi), vegetable protein beverages that were not soya-bean milk (e.g., soya-bean milk drink, walnut drink, syrup of almond), carbonated beverages (e.g., cola, Sprite), tea beverages (e.g., iced tea, jasmine tea), and sports and energy beverages (referred to as sports/energy beverages, e.g., Vitamin Water, Red Bull, Mizone) [20,30]. We categorized each beverage based on its nutrient content and China’s General Standard for Beverages (GB/T 10789-2015) [31]. The test–retest reliability of the food frequency questionnaire was good in the pilot study among a convenience sample of 175 caregivers, and the results are available in the previous paper [27]. The SSBs were deemed to be “consumed” when the consumption frequency for any category of SSBs mentioned above was other than “never”.

#### 2.2.2. Other Measures

For the children’s exposure to SSB-related obesogenic environments, we included the caregivers’ frequencies of SSB consumption, reading behaviors for the Nutrition Facts Panel (NFP), and frequencies of taking children to fast-food restaurants, as well as children’s exposures to SSB advertisements.

Children’s exposure frequencies to advertisements for the above six SSB categories were assessed with the question, “Usually in a week, how often was your child exposed (see or hear) to the advertisement for the following SSBs in the past three months? (including on the roadside, in kindergartens, in the community, on TV and the Internet, etc.)”, with the five Likert levels ranging from “never” to “more than once per day” and “not clear”. The responses were categorized into four levels (never, less than once per week, once to six times per week, or at least once per day). “Not clear” was considered as a missing value.

We measured the caregivers’ frequencies of SSB consumption using a seven-category scale with the question, “How often did you (caregivers) drink SSBs (such as carbonated beverages, sports beverages, but excluding freshly squeezed juices) in the past three months?” The response options were categorized into two levels (less than once per week or at least once per week) using dichotomy cutoffs.

The caregivers’ frequencies of NFP reading were assessed with the question, “How often did you (caregivers) read the Nutrition Facts Panel before buying food in the past three months?” The response options to these questions were “never”, “rarely”, “sometimes”, “most of the time”, and “always”. The variable was dichotomized into infrequently (never, rarely, or sometimes) or frequently (most of the time or always).

We assessed the caregivers’ frequencies of taking children to fast-food restaurants with the question, “Did you (caregivers) often take your child to fast-food restaurants (McDonald’s, KFC, Pizza Hut, Burger King, etc.) in the past three months?” We used a five-category scale ranging from “never or less than once per week” to “everyday”, and the response options were categorized into two levels (less than once per week or at least once per week) using dichotomy cutoffs.

For covariates, trained school nurses measured each child’s weight and height, their body mass index was calculated, and their weight status was defined using criteria developed by the World Health Organization to ensure the authenticity and comparability of the data [32,33,34]. Data on the child’s birth date, gender, family income, and whether the child was the only child in the family were also collected using questionaries. Family income based on the “annual household income per capita” item of this study and ranging from “50 thousand RMB/year or below” to “more than 150 thousand RMB/year” was used as a proxy for socio-economic status [17,22,24].

### 2.3. Statistical Analysis

Statistical analyses were completed using IBM SPSS Statistics for Windows (Version 25.0. Armonk, NY, USA: IBM Corp). Children’s characteristics were analyzed using descriptive statistics. Normally distributed variables were expressed as means ± standard deviations. Categorical variables were presented as frequency values and percentages, and the chi-square test was used for comparison between groups. The associations between the exposure to obesogenic environments and the consumption of SSBs were tested using binary logistic regression models, adjusting for the preschooler’s gender, age, whether the child was the only child in the family, and annual household income per capita. Statistical significance was considered as *p* ≤ 0.05 (two-sided).

## 3. Results

### 3.1. Characteristics of Participants

A total of 3057 caregivers participated in the survey, with a responding rate of 85.0% (3057/3595), and all of their data were available and included in the analysis. The caregiver was the study child’s parent (97.8%), grandparent (1.6%), or significant other (0.6%). The characteristics of the children according to their SSB consumption are described in Table 1. Among the 3057 children, the mean age was 5.6 ± 0.6 years. Nearly half (48.3%) were girls, 68.9% were the only child in the family, 15.2% were either overweight (10.4%) or obese (4.8%), and more than half (52.9%) of came from families with an annual household income per capita of more than 150 thousand RMB ($23,265). About 84.5% of the children had consumed SSBs over the past three months; children who were non-consumers of SSBs were more likely to have an annual household income per capita of more than 150 thousand RMB, and were less likely to be overweight or obese than those in the SSB consumption categories (all *p* < 0.05).

### 3.2. SSB Consumption Frequency

**Figure 1:**
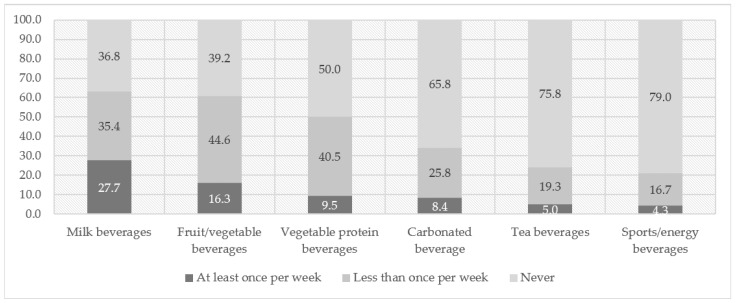
The proportions of sugar-sweetened beverage consumption over the past three months among preschoolers (%). Notes: Milk beverages (sugar-sweetened milk beverages that were not milk or yogurt, e.g., lactobacillus beverages, Nutri-Express, AD Calcium Milk); fruit/vegetable beverages (vegetable- or fruit-flavored beverages that were not 100 % fruit or vegetable juice, e.g., Orange Multi); vegetable protein beverages (e.g., soya-bean milk drink, walnut drink, syrup of almond); carbonated beverages (e.g., cola, Sprite); tea beverages (e.g., iced tea, jasmine tea); sports/energy beverages (e.g., Vitamin Water, Red Bull, Mizone).

Overall, over four-fifths (84.5%) of the preschoolers had consumed SSBs in the past three months. Milk beverages were the most-consumed SSB category (63.2%), followed by fruit/vegetable beverages (60.8%), vegetable protein beverages (50.0%), carbonated beverages (34.2%), tea beverages (24.2%), and sports/energy beverages (21.0%) (Figure 1).

### 3.3. Frequencies of Exposure to SSB-Related Obesogenic Environments

Children were exposed to milk beverage advertisements most frequently, with 17.0% being exposed more than once per day and 49.0% more than once per week, subsequently followed by carbonated beverages and fruit/vegetable beverages (Figure S1). In addition, about half of the caregivers consumed SSBs at least once per week (52.9%), more than one-third read NFPs frequently (37.7%), and about one-fifth took children to fast-food restaurants at least once per week (21.7%). The proportions of SSB consumption among preschoolers were significantly higher for children exposed to SSB advertisements and whose caregivers frequently consumed SSBs, took them to fast-food restaurants, and read NFPs infrequently (all *p* < 0.05) (Tables S1–S6).

### 3.4. Associations between Obesogenic Environments and SSB Consumption among Preschoolers

As shown in Table 2, higher exposure to advertisements of the corresponding SSB category by children, higher frequencies of consuming SSBs and taking children to fast-food restaurants by caregivers, and lower frequencies of reading the NFPs by caregivers were associated with higher consumption of SSBs among children. Similar trends of association were shown in all the SSB categories we investigated. Taking milk beverages as an example, after adjusting for potential confounders, children who were exposed to milk beverage advertisements “less than once per week” (*OR:* 2.123, 95% *CI:* 1.670–2.697), “1 to 6 times per week” (*OR:* 3.216, 95% *CI:* 2.503–4.134), or “at least once per day” (*OR:* 4.082, 95% *CI:* 3.038–5.485) were more likely to consume milk beverages compared with the “never” group. When caregivers consumed SSBs “at least once per week” (*OR*: 1.912, 95% *CI*: 1.623–2.251), their children were more likely to consume milk beverages. When taken to fast-food restaurants “at least once per week” (*OR*: 1.745, 95% *CI*: 1.411–2.157) by caregivers, the children were more likely to consume milk beverages compared with the “less than once per week” group. In contrast, the children whose caregivers frequently read the NFPs were less likely to consume milk beverages (*OR*: 0.579, 95% *CI*: 0.491–0.683) than the “infrequently” group.

## 4. Discussion

In this cross-sectional study among preschoolers in the Dongcheng District of Beijing, China, we discovered that more than four-fifths of preschoolers consumed SSBs over the past three months, which was associated with a higher risk of overweight and obesity. Children with more exposure to SSB advertisements consumed more SSBs. Additionally, parental behaviors including consuming SSBs, lack of NFP reading, and taking children to fast-food restaurants were associated with a higher likelihood of children’s SSB consumption. Overall, our findings suggest that decreasing child-targeted SSB advertising exposure, increasing parental nutritional labeling reading, and reducing parental SSB consumption and fast-food restaurant visits could be potential strategies to reduce children’s SSB consumption.

The present estimates of children’s SSB consumption seem to be in line with those in previous studies in China. For example, a study found that 79.5% of children aged three to seven years consumed SSBs during the preceding half-year in seven large cities in 2012, while they investigated only carbonated beverages, fruit/vegetable beverages, tea beverages, and sports/energy beverages [20]. The substantial evidence demonstrates that preschool is a crucial period for developing obesity and consequently metabolic risk [2,35]. The high prevalence rates of SSB consumption in this study and others support the need for urgent interventions focusing on SSB marketing and parental behaviors in obesogenic environments to promote healthy beverage consumption among preschoolers. In addition, our results indicated that the most frequently consumed SSBs were milk beverages, followed by fruit/vegetable beverages and vegetable protein beverages, broadly consistent with the existing evidence among urban preschoolers reported in previous studies [36]. In particular, the high consumption of milk beverages may be explained by confusion about milk and milk beverages in parental perceptions because of the lack of clear and truthful labeling of milk beverages to enable caregivers to make well-informed decisions [37,38,39]. Different studies reported that carbonated beverages were also preferred in Western countries instead of vegetable protein beverages [6]. Our findings suggest that for interventions an emphasis should probably be placed on milk beverages, fruit/vegetable beverages, and vegetable protein beverages in urban China.

The present study showed that caregivers’ rates of SSB consumption [24,40] and taking children to fast-food restaurants [41,42,43] frequently were positively associated with SSB consumption among their children, consistent with previous studies at home and abroad. Our study also identified that as low as one-third of caregivers read the NFPs frequently, which was negatively associated with SSB consumption among their children. The above behaviors of caregivers may be explained by their health literacy, which was associated with SSB consumption in both parents and children [44], as parents are the purchasing decision-makers for themselves and their children in obesogenic environments. In obesogenic environments, fast-food restaurants are important sources of SSBs and SSB marketing, with various SSB options, as well as a large volume of price promotion advertisements and child-directed advertisements with cartoon, TV, or movie characters [45]. In addition, previous studies have suggested that a lack of nutritional information restricted the efficacy of parents in making healthier SSB choices for their children at fast-food restaurants [46,47]. Additionally, the behavior of NFP reading might reflect caregivers’ self-efficacy in avoiding unhealthy foods and drinks in general. So far, few SSB products high in sugar have provided sugar content information on the NFP in China [48]. Nutritional labeling is a vital tool to promote healthier choices, while the previous evidence has suggested that compulsorily added new nutritional information (e.g., added sugars and percent daily values for added sugars) alone would not be sufficient to affect choices of beverages [49,50], and consumers might be required to improve their perceptions on healthy choices and their capability to interpret the information in nutritional labeling via health education and promotion, so as to use it frequently [51]. To reduce the SSB consumption rates among preschoolers, the use of evidence-based measures aiming to improve parental awareness of the health effects of SSBs and to improve their self-efficacy to choose healthier drinks [52,53,54,55] may be an indispensable strategy.

We observed positive associations between SSB advertisement exposure and consumption among preschoolers, consistent with the results of previous studies in other countries showing that SSB advertising strongly influences the long-term beverage preferences and consumption [44,56]. The elements of advertisements that influence their effectiveness include the extent of exposure to the message and the persuasive power of the communications related to the message design and content [57]. Our study showed that preschoolers were exposed to SSB advertisements frequently. The frequent occurrence of these marketing messages might influence social norms and promote the perception that calorie-dense, nutrient-poor beverages are normal [58,59,60]. In addition, the substantial evidence indicated that a significant portion of SSB advertising is typically aimed directly at children and parents [61,62,63]. The consumption demands of children and parents were promoted by the persuasive marketing techniques, including the following aspects: (i) promotional characters (e.g., cartoon figures and celebrities); (ii) themes of taste; (iii) the emotional appeal of fun; (iv) brand benefit claims; (v) brand health claims [64]. As a result, advertising misleads children and parents who lack media literacy [65]. Hence, legislation relating to mandatory regulations for SSB advertising to children across all kinds of media and marketing techniques is needed in China [66,67].

The findings of this study provide a timely update on the current evidence of SSB consumption and potential modifiable obesogenic environment determinants at the micro- and macro-levels among preschoolers, who are in a critical period in the life course and have been rarely studied in China. Meanwhile, the following limitations should be considered when interpreting this study. First, data obtained from self-reported questionnaires may be subject to recall bias and reporting bias, which may lead to underestimating the frequencies of SSB consumption and the exposure to obesogenic environments. We reduced the bias via unified training for the investigators, the use of an institution-based survey, and the use of anonymous questionnaires to establish close cooperation with the participants. Second, there are some other approaches for children to be exposed to obesogenic environments, such as children’s exposure to SSBs in kindergartens, which has been an important channel over a long period [68]. However, the Chinese government has released the 2021 Guide for the Construction of Nutrition and Health Schools, which requires food shops, supermarkets, and others not to be set up in kindergartens. Thus, these settings are not major settings for preschool students to access SSBs. Third, the Dongcheng district is in downtown Beijing, representing an advanced socioeconomic environment, which may restrict the generalization of the findings to broad contexts. Nevertheless, the current findings could provide vital references for other areas. Fourth, a cross-sectional study failed to infer causality for potential confounders. As the present analysis was exploratory and not intended to be a predictive model, we adjusted for key demographic characteristics; additional studies are required to confirm the results.

## 5. Conclusions

Exposures to SSB-related obesogenic environments, such as caregivers’ SSB consumption and taking children to fast-food restaurants, as well as children’s exposure to SSB advertisements, were associated with a higher likelihood of children’s SSB consumption. In contrast, caregivers’ NFP reading behaviors when buying foods and drinks were negatively associated with SSB consumption. As preschool age is a critical period for developing obesity and consequently metabolic risk that can possibly last a lifetime, targeting of SSB advertising as a part of efforts to reduce children’s SSB consumption is needed. Parents are the purchasing decision-makers for themselves and their children, and our results support the need for future health education and the promotion of parental self-efficacy and behavior to avoid SSBs in obesogenic environments.

## Figures and Tables

**Table 1:** Basic characteristics of the surveyed preschoolers and their sugar-sweetened beverage (SSB) consumption over the past three months: *n* (%).

Characteristics	Total (*n* = 3057)	SSB Consumption among Preschoolers			
		Yes (*n* = 2583)	No (*n* = 474)	*χ*^2^/*t*	*p* Value
**Gender**				0.159	0.690
Boys	1580 (51.7)	1339 (84.7)	241 (15.3)		
Girls	1477 (48.3)	1244 (84.2)	233 (15.8)		
**Age (years) groups**				5.425	0.066
≤5	579 (18.9)	472 (81.5)	107 (18.5)		
>5 and ≤6	1664 (54.4)	1411 (84.8)	253 (15.2)		
>6	814 (26.6)	700 (86.0)	114 (14.0)		
**Whether the only child in the family**				1.764	0.184
No	950 (31.1)	815 (85.8)	135 (14.2)		
Yes	2107 (68.9)	1768 (83.9)	339 (16.1)		
**Annual household income per capita**				13.811	<0.001
≤150 thousand RMB (23,265 dollars)	1439 (47.1)	1253 (87.1)	186 (12.9)		
>150 thousand RMB	1618 (52.9)	1330 (82.2)	288 (17.8)		
**Weight status**				12.312	0.002
Underweight or normal-weight	2593 (84.8)	2166 (83.5)	427 (16.5)		
Overweight	318 (10.4)	284 (89.3)	34 (10.7)		
Obese	146 (4.8)	133 (91.1)	13 (8.9)		

**Table 2:** Associations between sugar-sweetened beverages (SSBs) consumption over the past three months and exposure to obesogenic environments among preschoolers: multivariate analysis.

**Variable**	Milk Beverages *		**Fruit/Vegetable** Beverages *		Vegetable Protein Beverages *	
	***OR* (95% *CI*)**	***p* Value**	***OR* (95% *CI*)**	***p* Value**	***OR* (95% *CI*)**	***p* Value**
**Girls** (reference: boys)	1.101 (0.937–1.294)	0.241	0.959 (0.818–1.125)	0.606	0.860 (0.738–1.002)	0.053
**Age group** (years)						
>5 and ≤6 (reference: ≤5)	0.891 (0.718–1.106)	0.296	1.028 (0.831–1.271)	0.799	1.002 (0.816–1.230)	0.987
>6 (reference: ≤5)	0.920 (0.720–1.175)	0.502	1.078 (0.847–1.372)	0.541	1.306 (1.035–1.647)	0.025
**The only child in the family** (reference: no)	0.823 (0.690–0.981)	0.030	0.775 (0.651–0.922)	0.004	0.933 (0.791–1.100)	0.409
**Annual household income per capita >150 thousand RMB** (23,265 dollars) (reference: ≤150 thousand RMB)	0.852 (0.725–1.002)	0.053	0.753 (0.642–0.884)	<0.001	0.734 (0.630–0.856)	<0.001
**Exposure to advertisements of the corresponding SSB category** (reference: never)						
Less than once per week	2.123 (1.670–2.697)	<0.001	4.027 (3.194–5.077)	<0.001	2.876 (2.341–3.534)	<0.001
1 to 6 times per week	3.216 (2.503–4.134)	<0.001	5.346 (4.163–6.866)	<0.001	4.498 (3.541–5.713)	<0.001
At least once per day	4.082 (3.038–5.485)	<0.001	4.646 (3.447–6.261)	<0.001	3.723 (2.773–4.998)	<0.001
**Caregivers consumed SSBs at least once per week** (reference: less than once per week)	1.912 (1.623–2.251)	<0.001	1.527 (1.299–1.796)	<0.001	1.210 (1.035–1.415)	0.017
**Caregivers read the Nutrition Facts Panel frequently** (reference: infrequently)	0.579 (0.491–0.683)	<0.001	0.681 (0.578–0.803)	<0.001	0.852 (0.727–0.999)	0.049
**Caregivers took children to fast-food restaurants at least once per week** (reference: less than once per week)	1.745 (1.411–2.157)	<0.001	1.608 (1.310–1.973)	<0.001	1.420 (1.176–1.716)	<0.001
**Variable**	Carbonated beverages *		Tea beverages *		**Sports/energy beverages** *	
	***OR* (95% *CI*)**	***p* value**	***OR* (95% *CI*)**	***p* value**	***OR* (95% *CI*)**	***p* value**
**Girls** (reference: boys)	0.838 (0.714–0.983)	0.030	0.866 (0.723–1.037)	0.118	0.625 (0.516–0.756)	<0.001
**Age group** (years)						
>5 and ≤6 (reference: ≤5)	1.183 (0.950–1.474)	0.133	1.163 (0.904–1.497)	0.241	1.288 (0.977–1.700)	0.073
>6 (reference: ≤5)	1.629 (1.277–2.078)	<0.001	1.555 (1.180–2.050)	0.002	1.975 (1.469–2.655)	<0.001
**The only child in the family** (reference: no)	0.754 (0.635–0.895)	0.001	0.831 (0.685–1.007)	0.059	0.698 (0.573–0.851)	<0.001
**Annual household income per capita >150 thousand RMB** (23,265 dollars) (reference: ≤150 thousand RMB)	0.998 (0.850–1.171)	0.979	0.737 (0.616–0.883)	<0.001	0.840 (0.696–1.014)	0.069
**Exposure to advertisements of the corresponding SSB category** (reference: never)						
Less than once per week	2.695 (2.095–3.465)	<0.001	4.564 (3.393–6.139)	<0.001	3.674 (2.769–4.875)	<0.001
1 to 6 times per week	3.270 (2.517–4.248)	<0.001	5.968 (4.349–8.190)	<0.001	4.120 (3.007–5.644)	<0.001
At least once per day	3.142 (2.330–4.238)	<0.001	5.211 (3.624–7.494)	<0.001	4.257 (2.961–6.119)	<0.001
**Caregivers consumed SSBs at least once per week** (reference: less than once per week)	1.716 (1.454–2.025)	<0.001	1.714 (1.420–2.068)	<0.001	1.508 (1.239–1.836)	<0.001
**Caregivers read the Nutrition Facts Panel frequently** (reference: infrequently)	0.720 (0.608–0.852)	<0.001	0.700 (0.577–0.848)	<0.001	0.845 (0.693–1.030)	0.096
**Caregivers took children to fast-food restaurants at least once per week** (reference: less than once per week)	1.661 (1.376–2.005)	<0.001	1.825 (1.489–2.238)	<0.001	1.647 (1.331–2.037)	<0.001

Notes: Adjusted for the preschooler’s gender, age, whether the child was the only child in the family, annual per capita family income. * Milk beverages (sugar-sweetened milk beverages that were not milk or yogurt, e.g., lactobacillus beverages, Nutri-Express, AD Calcium Milk); fruit/vegetable beverages (vegetable- or fruit-flavored beverages that were not 100 % fruit or vegetable juice, e.g., Orange Multi); vegetable protein beverages (e.g., soya-bean milk drink, walnut drink, syrup of almond); carbonated beverages (e.g., cola, Sprite); tea beverages (e.g., iced tea, jasmine tea); sports/energy beverages (e.g., Vitamin Water, Red Bull, Mizone).

## Data Availability

According to private and confidential clauses stated in the informed consent, the raw data supporting the conclusions of this article are ethically restricted and not publicly available. They would be available from Prof. Juan ZHANG (E-mail: zhangjuan@sph.pumc.edu.cn) on reasonable request.

## Supplementary Materials

The following are available online at https://www.mdpi.com/article/10.3390/nu14142860/s1. Figure S1: Frequencies of exposure to SSB advertisements among preschoolers (%). Table S1: Associations between milk beverage consumption and exposure to obesogenic environments among preschoolers: univariate analysis: *n* (%). Table S2: Associations between fruit/vegetable beverage consumption and exposure to obesogenic environments among preschoolers: univariate analysis: *n* (%). Table S3: Associations between vegetable protein beverage consumption and exposure to obesogenic environments among preschoolers: univariate analysis: *n* (%). Table S4: Associations between carbonated beverage consumption and exposure to obesogenic environments among preschoolers: univariate analysis: *n* (%). Table S5: Associations between tea beverage consumption and exposure to obesogenic environments among preschoolers: univariate analysis: *n* (%). Table S6: Associations between sports/energy beverage consumption and exposure to obesogenic environments among preschoolers: univariate analysis: *n* (%).

## Abbreviations

SSBs: sugar-sweetened beverages; NFP: Nutrition Facts Panel.
