# Supplementary material for: Impact of Obesogenic Environments on Sugar-Sweetened Beverage Consumption among Preschoolers: Findings from a Cross-Sectional Survey in Beijing
Source: Nutrients. 2022 Jul 12;14(14):2860. doi: 10.3390/nu14142860 (PMC9321344; doi:10.3390/nu14142860)
Supplement: Supplementary file 1 [file nutrients-14-02860-s001.zip › nutrients-1784163-supplementary.pdf]

Supplementary Materials

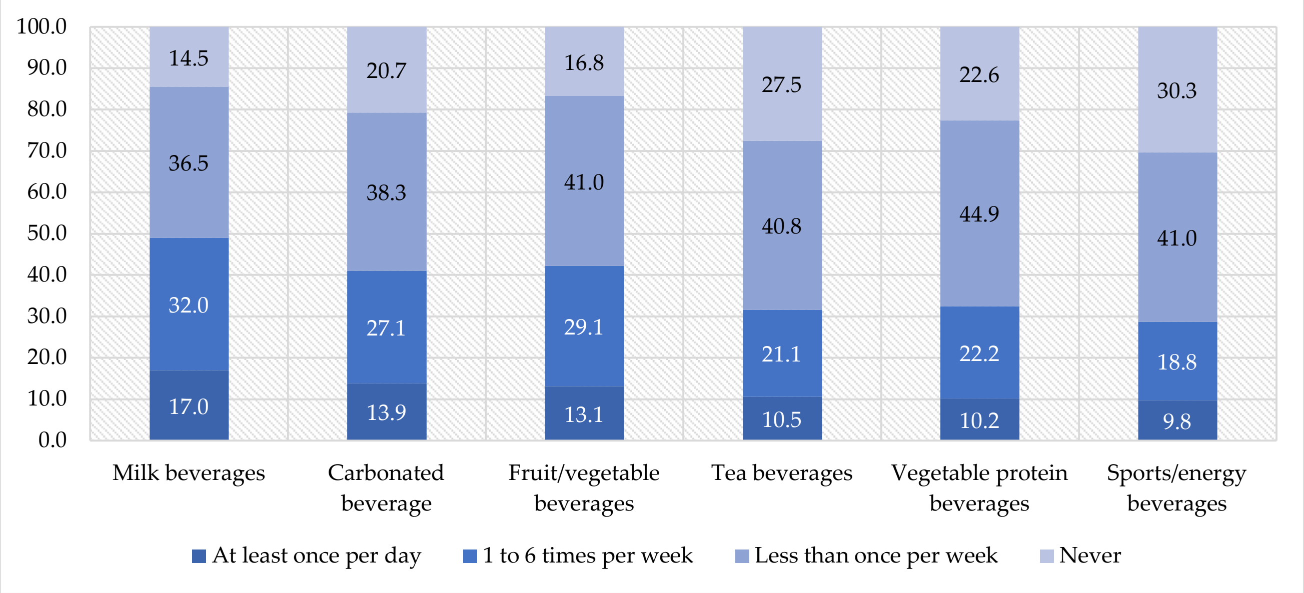

Figure S1. Frequencies of exposure to sugar-sweetened beverage advertisement among preschoolers (%).

Notes: Milk beverages (sugar-sweetened milk beverages that were not milk or yogurt, e.g., lactobacillus beverages, Nutri-Express, AD Calcium Milk); fruit/vegetable beverages (vegetable- or fruit-flavored beverages that were not 100 % fruit or vegetable juice, e.g., Orange Multi); vegetable protein beverages (e.g., soya-bean milk drink, walnut drink, syrup of almond); carbonated beverages (e.g., cola, Sprite); tea beverages (e.g., iced tea, jasmine tea); sports/energy beverages (e.g., Vitamin Water, Red Bull, Mizone).

Table S1. Associations between milk beverages consumption and exposure to obesogenic environments among preschoolers: Univariate analysis: *n* (%)

**Variables**	**Total**	**Milk beverages consumption among preschoolers**			
		Yes	No	χ^2/t	*P* value
**Milk beverage advertisements exposure among preschoolers (*n* = 2875)**				155.411	< 0.001
Never	417 (14.5)	169 (40.5)	248 (59.5)		
Less than once per week	1048 (36.5)	632 (60.3)	416 (39.7)		
1 to 6 times per week	921 (32.0)	654 (71.0)	267 (29.0)		
At least once per day	489 (17.0)	372 (76.1)	117 (23.9)		
**Caregivers took children to fast-food restaurants**				60.099	< 0.001
Less than once per week	2394 (78.3)	1427 (59.6)	967 (40.4)		
At least once per week	663 (21.7)	504 (76.0)	159 (24.0)		
**Caregivers read the Nutrition Facts Panel**				62.176	< 0.001
Infrequently	1903 (62.3)	1304 (68.5)	599 (31.5)		
Frequently	1154 (37.7)	627 (54.3)	527 (45.7)		
**Caregivers consumed sugar-sweetened beverage**				121.888	< 0.001
Less than once per week	1439 (47.1)	762 (53.0)	677 (47.0)		
At least once per week	1618 (52.9)	1169 (72.2)	449 (27.8)		

Table S2. Associations between fruit/vegetable beverages consumption and exposure to obesogenic environments among preschoolers: Univariate analysis: *n* (%)

Variables	Total	Fruit/vegetable beverages consumption among preschoolers			
		Yes	No	χ^2/t	*P* value
**Fruit/vegetable beverage advertisements exposure among preschoolers (*n*=2901)**				244.430	<0.001
Never	486 (16.8)	339 (69.8)	147 (30.2)		
Less than once per week	1190 (41.0)	428 (36.0)	762 (64.0)		
1 to 6 times per week	844 (29.1)	244 (28.9)	600 (71.1)		
At least once per day	381 (13.1)	118 (31.0)	263 (69.0)		
**Caregivers took children to fast-food restaurants**				47.441	<0.001
Less than once per week	2394 (78.3)	1014 (42.4)	1380 (57.6)		
At least once per week	663 (21.7)	183 (27.6)	480 (72.4)		
**Caregivers read the Nutrition Facts Panel**				33.873	<0.001
Infrequently	1903 (62.3)	669 (35.2)	1234 (64.8)		
Frequently	1154 (37.7)	528 (45.8)	626 (54.2)		
**Caregivers consumed sugar-sweetened beverage**				68.571	<0.001
Less than once per week	1439 (47.1)	675 (46.9)	764 (53.1)		
At least once per week	1618 (52.9)	522 (32.3)	1096 (67.7)		

Table S3. Associations between vegetable protein beverages consumption and exposure to obesogenic environments among preschoolers: Univariate analysis: *n* (%)

Variables	Total	Vegetable protein beverages consumption among preschoolers			
		Yes	No	χ^2/t	*P* value
**Vegetable protein beverage advertisements exposure among preschoolers (*n* = 2899)**				204.010	<0.001
Never	656 (22.6)	477 (72.7)	179 (27.3)		
Less than once per week	1303 (44.9)	616 (47.3)	687 (52.7)		
1 to 6 times per week	645 (22.2)	230 (35.7)	415 (64.3)		
At least once per day	295 (10.2)	118 (40.0)	177 (60.0)		
**Caregivers took children to fast-food restaurants**				29.990	<0.001
Less than once per week	2394 (78.3)	1259 (52.6)	1135 (47.4)		
At least once per week	663 (21.7)	269 (40.6)	394 (59.4)		
**Caregivers read the Nutrition Facts Panel**				6.508	0.011
Infrequently	1903 (62.3)	917 (48.2)	986 (51.8)		
Frequently	1154 (37.7)	611 (52.9)	543 (47.1)		
**Caregivers consumed sugar-sweetened beverage**				24.096	<0.001
Less than once per week	1439 (47.1)	787 (54.7)	652 (45.3)		
At least once per week	1618 (52.9)	741 (45.8)	877 (54.2)		

Table S4. Associations between carbonated beverages consumption and exposure to obesogenic environments among preschoolers: Univariate analysis: *n* (%)

Variables	Total	Carbonated beverages consumption among preschoolers			
		Yes	No	χ^2/t	*P* value
**Carbonated beverage advertisements exposure among preschoolers (*n* = 2907)**				119.611	<0.001
Never	603 (20.7)	502 (83.3)	101 (16.7)		
Less than once per week	1112 (38.3)	711 (63.9)	401 (36.1)		
1 to 6 times per week	789 (27.1)	451 (57.2)	338 (42.8)		
At least once per day	403 (13.9)	233 (57.8)	170 (42.2)		
**Caregivers took children to fast-food restaurants**				63.847	<0.001
Less than once per week	2394 (78.3)	1662 (69.4)	732 (30.6)		
At least once per week	663 (21.7)	350 (52.8)	313 (47.2)		
**Caregivers read the Nutrition Facts Panel**				24.155	<0.001
Infrequently	1903 (62.3)	1190 (62.5)	713 (37.5)		
Frequently	1154 (37.7)	822 (71.2)	332 (28.8)		
**Caregivers consumed sugar-sweetened beverage**				78.399	<0.001
Less than once per week	1439 (47.1)	1063 (73.9)	376 (26.1)		
At least once per week	1618 (52.9)	949 (58.7)	669 (41.3)		

Table S5. Associations between tea beverages consumption and exposure to obesogenic environments among preschoolers: Univariate analysis: *n* (%)

Variables	Total	Tea beverages consumption among preschoolers			
		Yes	No	χ^2/t	*P* value
**Tea beverage advertisements exposure among preschoolers (*n* = 2893)**				185.918	<0.001
Never	797 (27.5)	737 (92.5)	60 (7.5)		
Less than once per week	1181 (40.8)	849 (71.9)	332 (28.1)		
1 to 6 times per week	610 (21.1)	392 (64.3)	218 (35.7)		
At least once per day	305 (10.5)	204 (66.9)	101 (33.1)		
**Caregivers took children to fast-food restaurants**				67.615	<0.001
Less than once per week	2394 (78.3)	1894 (79.1)	500 (20.9)		
At least once per week	663 (21.7)	422 (63.7)	241 (36.3)		
**Caregivers read the Nutrition Facts Panel**				21.878	<0.001
Infrequently	1903 (62.3)	1388 (72.9)	515 (27.1)		
Frequently	1154 (37.7)	928 (80.4)	226 (19.6)		
**Caregivers consumed sugar-sweetened beverage**				71.220	<0.001
Less than once per week	1439 (47.1)	1190 (82.7)	249 (17.3)		
At least once per week	1618 (52.9)	1126 (69.6)	492 (30.4)		

Table S6. Associations between sports/energy beverages consumption and exposure to obesogenic environments among preschoolers: Univariate analysis: *n* (%)

Variables	Total	Sports/energy beverages consumption among preschoolers			
		Yes	No	χ^2/t	*P* value
**Sports/energy beverage advertisements exposure among preschoolers (*n* = 2897)**				135.587	<0.001
Never	879 (30.3)	810 (92.2)	69 (7.8)		
Less than once per week	1188 (41.0)	889 (74.8)	299 (25.2)		
1 to 6 times per week	546 (18.8)	390 (71.4)	156 (28.6)		
At least once per day	284 (9.8)	200 (70.4)	84 (29.6)		
**Caregivers took children to fast-food restaurants**				44.285	<0.001
Less than once per week	2394 (78.3)	1953 (81.6)	441 (18.4)		
At least once per week	663 (21.7)	462 (69.7)	201 (30.3)		
**Caregivers read the Nutrition Facts Panel**				5.393	0.020
Infrequently	1903 (62.3)	1478 (77.7)	425 (22.3)		
Frequently	1154 (37.7)	937 (81.2)	217 (18.8)		
**Caregivers consumed sugar-sweetened beverage**				35.743	<0.001
Less than once per week	1439 (47.1)	1204 (83.7)	235 (16.3)		
At least once per week	1618 (52.9)	1211 (74.8)	407 (25.2)		
